# Malta: Considered with Respect to Its Eligibility as a Place of Residence for Invalids

**Published:** 1841-04-01

**Authors:** 


MALTA: CONSIDERED WITH REFERENCE TO ITS ELIGIBILITY AS A PLACE OF RESIDENCE FOR INVALIDS. Addressed to the Members of the Medical Profession. By *Francis Sankey*, M.D.

FIRST impressions, especially if strong, and on young minds, are rarely expunged by those of later years, however erorneous may be the first, or correct the subsequent ones. This may be the case in respect to writers on Malta. Byron designated it as a "MILITARY HOTHOUSE," probably under the influence of momentary spleen or misanthropy. Hennen, the sober and philosophic topographist—our old and esteemed friend—was not more than mortal, and had his prejudices and predilections, when he wrote about "the implacable heat;" and "the showers of mud" in the Island of Malta. In respect to temperature, the highest range of the thermometer in the two hottest months, July and August, is 82°, whilst in the eight months that include October and May, the range is from 53° to 70°.

The island lies nearly in the centre of the Mediterranean, and almost within sight of Sicily. Although certain winds (the Siroc) are occasionally uncomfortable, in the Summer months, their inconvenience has been greatly exaggerated. The surface of the island is undulating, and does not present any hills higher than 600 feet above the level of the sea. There are neither woods nor marshes, and the orange and lemon plantations are enclosed within garden walls. The population is about 120,000 including the military. Valetta is a regular town, with wide and straight streets, crossing each other at right angles, and built on a small peninsula. The houses are commodious, the rooms large and lofty, provisions plentiful, and cheaper than in England. The inns are good and numerous. The thermometer does not vary more than four or five degrees during the twenty-four hours, from the end of September till the end of May, during which period the temperature is delightful—a complete Spring reigns in the Island. In February and March there are severe gusts of wind, which sweep freely over the Island; but are destitute of the keen cutting breezes that descend from the Alps or Appennines. The heat of the Summer, too, is moderated by cooling currents of air from the ocean, unobstructed by hills or forests. The effects of the Sirocco have been ridiculously magnified by Byron, and all who have written since his time. To it the fanciful ascribe all their morbid feelings; and the imprudent find a ready excuse for their indiscretions in the Siroc. In August and September this wind is often disagreeable, coming, as it does, both hot and humid from the south-east. In Africa it is a dry and hot wind, but becomes impregnated with moisture in its passage over the ocean. It seldom blows for more than a day, at one time, and in nervous people, causes languor and depression. In the Winter it is soft and balmy, and favourable to invalids with dry cough and bronchial irritation. The "showers of mud," described by Dr. Hennen, are merely the dust, which is sometimes rolled along by the wind, and which is occasionally united with aqueous moisture, as on the road-sides in England. The slight sources of malaria in Dr. Hennen's time have been all dried up. There are no vegetable matters running into a state of decomposition, nor animal matters either.

It appears from Major Tulloch's statistical reports, that Malta is not so healthy as Great Britain.

"This report is based on the deaths occurring in an average population of one hundred thousand people, compared with the same number in England. It is not just to ground such a statement on the deaths alone. The proper basis of the calculation would be the number of the sick; for such is the difference between the two countries, that a simple affection, which, in England, would be cured in a few days, is allowed by the natives to degenerate into serious disease. The people cannot pay for good medical attendance, and are averse to taking any sort of medicine. They have not the means of changing a scanty and unwholesome diet for more nourishing aliment. The people in the country eat the coarsest description of food, bad bread, crude vegetables, olives, and inferior kinds of salt fish. Meat they seldom touch, and wine only on festival days: and then perhaps to excess. The long fasting of forty-four days in Lent, in addition to the generally unhealthy course of diet, is another source of debility, and therefore, a predisposing cause of disease. From these circumstances, coupled with bad clothing and dirty habits, the people here have not sufficient stamina to support disease, so that, of an equal number of patients in England and Malta, a far greater proportion would recover in the former country. If due allowance be made for all the diseases which the inhabitants bring on, or continue on themselves, either by carelessness or necessity, there will not be found in the south of Europe a more healthy spot than this island. The mortality here, notwithstanding the causes above stated, cannot, in ordinary years, be calculated at so much as three per cent. The average number of deaths annually, according to the Statistical Report, for thirteen years, is 2,577, which, on an average population of 100,270, amounts to rather more than two and-a-half per cent." 13.

There is another error under the head of pectoral complaints. The Maltese practitioners apply the term consumption to " any and every wasting of the body from whatever cause it may arise;" even including the natural decay of old age. All surgical affections terminating by suppuration or hectic fever, are also classed under the head of phthisis! Thus the wife of a judge received a severe burn, and died of its effects. The case was returned consumption. It is stated that there is a greater mortality among the same number of troops in Malta than in England. This, however, may be attributed to other causes than climate alone. The facility of intoxication, and the propensity to that vice among soldiers and sailors, must greatly increase the mortality. The Fencible regiment of Maltese shews only a mortality of little more than one per cent. per annum, and this low degree can only be accounted for by the sober habits of the native soldiers. Civilians do not suffer near so much on the Island as the troops.

" I do not, of course, pretend to affirm, that the climate and air of Malta possess any curative virtue for those persons, whose organic diseases have placed them beyond the power of recovery elsewhere. Where tubercular disease has proceeded so far as to make extensive ravages in the pulmonary tissue, it is ever useless and cruel to send such unhappy persons far from their homes and sympathizing friends, to die among strangers in a foreign land.

At the present day it is pretty generally understood by medical practitioners, that such removal is mischievous, both from the fatigue attending the journey, and chiefly that the change to a warmer latitude, by increasing the irritability of the hectic sufferer, and augmenting the nocturnal perspirations, would farther reduce the strength of the patient, and hasten the catastrophe. Yet sometimes the medical attendant trusts that disease has not committed the ravages he suspects, or he is induced to yield to the wishes and solicitations of the patient, whose hope of recovery often appears commensurate with the fatal tendency of his complaint.

But to persons who are suffering from constitutional debility, who, either from conformation or accidental circumstances, are strongly predisposed to pulmonary disease, or are in any state of cachexia, unattended with considerable organic lesion, a residence in a southern latitude, for three or four months during the Winter is highly useful; and I think Malta offers advantages to such persons ,equal to those of any other place; amongst those advantages we may enumerate,—the voyage by sea, when land travelling would be too fatiguing: the facility of conveyance from any part of the surrounding Continents; the novelty of the scene, so different from any thing which Europe can offer: the living under the British flag, in a British possession: the many comforts that may be commanded: the power of obtaining the assistance of English medical practitioner residents in the island, as well as those of the staff of the army, or navy: and a climate where, on every day of the year, for some hours of that day, exercise in the open air, or gentle boat exercise, may be taken, so essential to the re-establishment of health." 20.

One other advantage is the facility of removal from Malta, should the climate disagree. Almost every year there are steamers leaving Malta for England, France, Italy, &c.

"The residence of her majesty the Queen Dowager Adelaide, during the Winter of 1838-9, has tended greatly to give a deserved notoriety to this island; and her liberality has added a handsome church to its public buildings." 21.

Upon the whole, we think it not improbable that Malta will become a rather favourable Winter asylum for English invalids. In the Summer, they can easily land at Genoa or Nice—skirt the Italian lakes—cross the Alps—traverse Switzerland—and descend the Rhine to their native soil.

